# MyACR: A Point-of-Care Medical Device for Determination of Albumin–Creatinine Ratio (uACR) in Random Urine Samples as a Marker of Nephropathy

**DOI:** 10.3390/diagnostics14161702

**Published:** 2024-08-06

**Authors:** Nadda Muhamad, Napaporn Youngvises, Tullayakorn Plengsuriyakarn, Wanchai Meesiri, Wanna Chaijaroenkul, Kesara Na-Bangchang

**Affiliations:** 1 Department of Biomedicine and Health Informatics, Faculty of Pharmacy, Silpakorn University, Nakhon Pathom 73000, Thailand; muhamad_n@su.ac.th; 2 Bangkok High Lab Co., Ltd., Bang Khen District, Bangkok 10220, Thailand; nyoungvises@hotmail.com (N.Y.); wanchai.meesiri@bangkokhighlab.com (W.M.); 3 Graduate Program in Bioclinical Sciences, Chulabhorn International College of Medicine, Thammasat University, Pathum Thani 12120, Thailand; tul_sheva@hotmail.com (T.P.); cwanna@tu.ac.th (W.C.)

**Keywords:** albumin, albumin-to-creatinine ratio, creatinine, microalbumin, point-of-care medical device

## Abstract

Chronic kidney disease (CKD) is a progressive condition that affects more than 10% of the world’s population. Monitoring urine albumin-to-creatinine ratio (uACR) has become the gold standard for nephropathy diagnosis and control. The objective of the present study was to develop a simple, accurate, sensitive, and rapid point-of-care test (PoCT) device, MyACR, for uACR measurement, intended for use in community healthcare to screen for the risk and monitor the progress of CKD. Albumin and creatinine concentrations in urine samples were determined using spectrophotometric dye (tetrabromophenol blue)-binding and colorimetric Jaffe assay, respectively. Urine samples were diluted with distilled water (1:80) and mixed separately with albumin and creatinine reaction mixture. The creatinine reaction was incubated at room temperature (25 °C) for 30 min before analysis. Optical density (OD) was measured at the wavelengths of 625 nm (albumin) and 515 nm (creatinine). All calibration curves (0–60 mg/L and 0–2 mg/dL for albumin and creatinine) yielded linear relationships with correlation coefficients (R^2^) of >0.997. Good accuracy (% deviation of mean value (DMV) ≤ 5.42%) and precision (% coefficients of variation (CV) ≤ 12.69%) were observed from both the intra- and inter-day assays for the determination of albumin and creatinine using MyACR. The limit of quantification (LOQ) of albumin and creatinine in urine samples determined using MyACR and a laboratory spectrophotometer were 5 mg/L and 0.25 mg/dL, respectively, using 37.5 μL urine spiked samples (*n* = 5). The device was well-applied with clinical samples from 20 CKD patients. The median (range) of %DMV of the central (hospital) laboratory method (immune-based assay) was 3.48 (−17.05 to 21.64)%, with a high correlation coefficient (R^2^ > 0.98). In conclusion, MyACR showed satisfactory test performance in terms of accuracy, reproducibility, and sensitivity. Cost-effectiveness and improvement in clinical decision making need to be proven in future multisite community and home studies.

## 1. Introduction

Nephropathy has increasingly become a significant global health concern in recent years [1,2]. Diabetes and hypertension are typically the primary causes of nephropathy, which can result in kidney damage, chronic kidney disease (CKD), renal failure, and mortality [3]. CKD is defined as either kidney damage or a glomerular filtration rate (GFR) < 60 mL/min per 1.73 m^2^ body surface area for three months or more, irrespective of cause, and stages of CKD severity are classified based on GFR [2,4,5]. The early stages of CKD are typically asymptomatic. As the condition progresses, however, patients may develop symptoms, such as nausea, vomiting, loss of appetite, fatigue, weakness, sleep disturbances, shortness of breath, lower back pain, and swelling in the feet and ankles [5]. Severe cases of CKD can lead to significant complications, such as ischemic heart disease, stroke, peripheral vascular disease, and cancer [2,6]. Early detection of nephropathy, especially in patients with diabetes and hypertension, is thus urgently required to prevent the progression of kidney damage.

Kidney function disorders and kidney disease are commonly evaluated through 24 h urine tests, in which urine is collected over a 24 h period to provide a precise evaluation of health status [7]. Although the determinations of creatinine and albumin from 24 h urine samples are considered the gold standard and widely used laboratory tests for kidney disorders, these tests pose challenges, including inconvenience for patients and potential low adherence to instructions. To address this limitation, the assessment of the albumin-to-creatinine ratio from random urine (uACR) serves as an alternative, as it is highly correlated with 24 h urine albumin [8,9,10,11,12]. Nephropathy occurs when the uACR is >30 mg/g creatinine or microalbuminuria is present (urinary albumin 30–300 mg/24 h) [13]. Without early detection and treatment, microalbuminuria may progress to irreversible macroalbuminuria (>300 mg/24 h) [14], which increases the risk of CKD progression and cardiovascular events.

The common methods that currently used for urine albumin and creatinine determination for the uACR assessment are immunoturbidimetry [15,16] and enzymatic assay [17,18], respectively, by using automated machine. However, these methods are costly, require advanced and complex instruments, and necessitate professional expertise, thus limiting their use at community health service centers. This emphasizes the need to develop highly sensitive point-of-care testing (PoCT) devices with simple techniques for rapid albuminuria tests to facilitate early CKD detection, monitoring, and management.

Hence, the objective of the present study was to develop a simple, accurate, sensitive, and rapid PoCT device—MyACR for uACR assessment—for simultaneous determination of urine creatinine and albumin. The clinical applicability of the device was validated in comparison with the gold standard test used in the central laboratory (immune-based assay) in urine samples obtained from CKD patients. Further study is required to evaluate its potential use by community healthcare professionals in resource-limited facilities and by patients in their own homes for screening the risk and monitoring the progress of CKD.

## 2. Materials and Methods

### 2.1. Chemicals and Reagents

Creatinine and tetrabromophenol blue (TBPB) were purchased from Sigma-Aldrich (St. Louis, MO, USA). Picric acid (PA) was obtained from Power Tech Chemical (Bangkok, Thailand). Human serum albumin (HSA) was purchased from Sisco Research Laboratories (Mumbai, India). Sodium sulfate anhydrous was purchased from QReC, New Zealand. Trisodium citrate dihydrate, ammonium chloride, magnesium sulfate heptahydrate, and disodium oxalate were obtained from Loba Chemie (Mumbai, India). Urea was purchased from Affymetrix (Cleveland, OH, USA). Potassium chloride and sodium chloride were obtained from Ajax Finechem (Wollongong, Australia). Sodium phosphate monobasic monohydrate was purchased from Carlo Erba (Emmendingen, Germany). Sodium phosphate dibasic heptahydrate and calcium chloride were purchased from Merck KGaA (Darmstadt, Germany). Sodium bicarbonate was obtained from VWR Life Science AMRESCO (Bridgeport, PA, USA).

A stock solution (20 mg/mL) of HSA and creatinine was prepared with deionized water and stored at 4 °C. The working solutions were obtained by diluting the stock solution with deionized water before use.

Artificial urine was prepared using the ingredients shown in Table 1 [19].

### 2.2. Instrumentation and Measurement Platform

**Figure 1:**
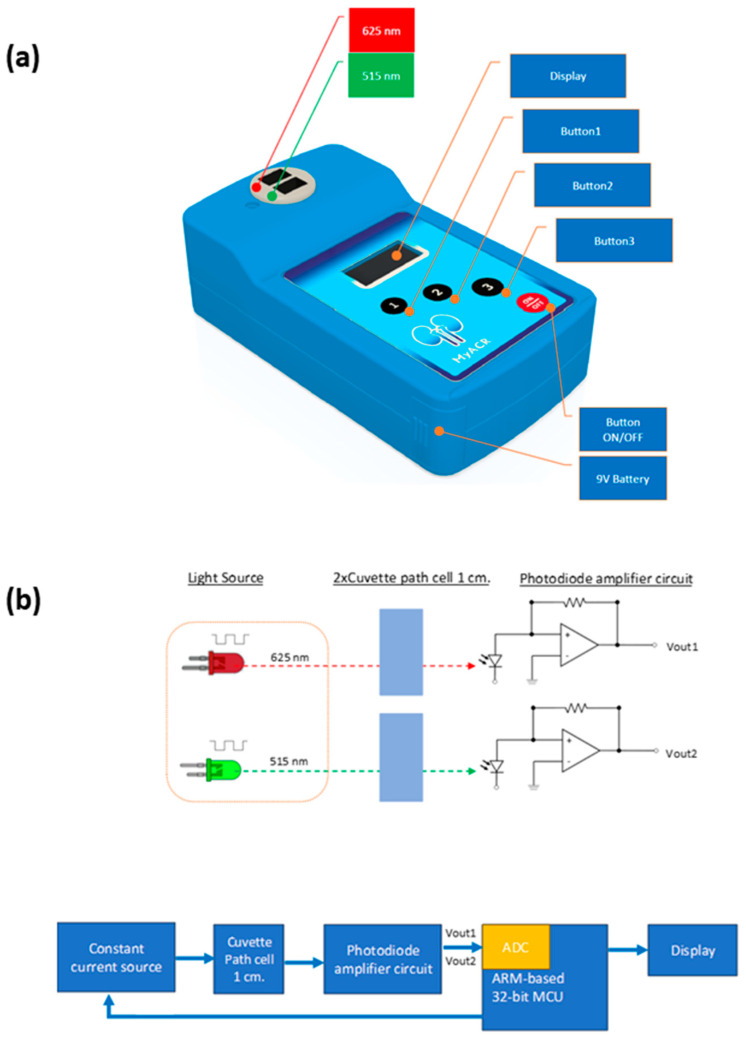
(**a**) MyACR device and (**b**) measurement platform.

**Figure 2:**
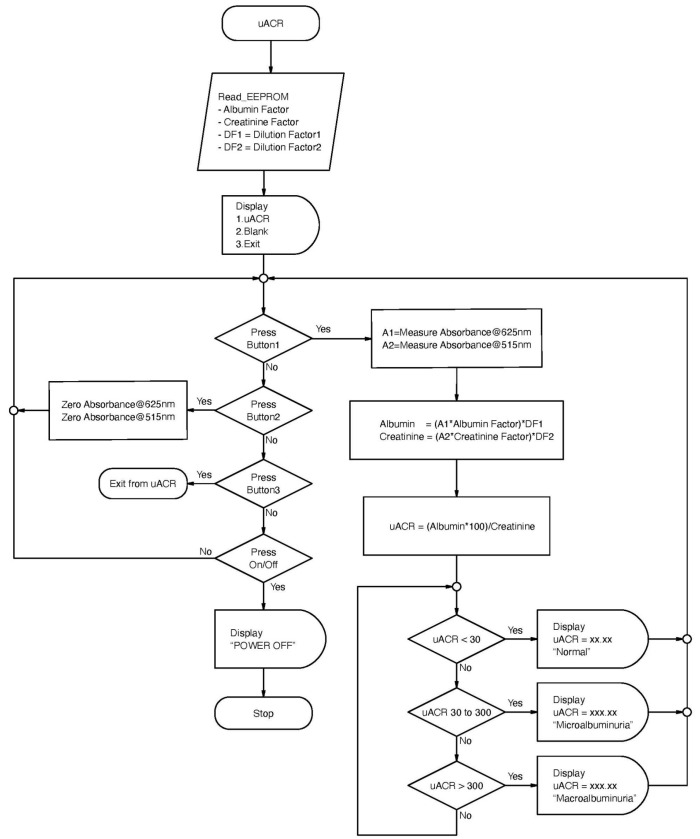
Schematic diagram showing the operational workflow of MyACR.

MyACR was developed as a colorimetric-based spectroscopic PoCT device to determine uACR utilizing a dual optical sensor. The test platform of the MyACR device is shown in Figure 1, and the schematic diagram of the system’s operation is shown in Figure 2. The MCU (microcontroller unit) sends a signal to the constant current source circuit to transmit UV light at 625 nm and 515 nm to pass through the cuvette and photodiode amplifier circuit. The detector receives V_out_-1 and V_out_-2 signals, converts them to ADC (analog-to-digital converter), and filters out only the desired frequency signals. The noise from outside ambient light is removed and used to calculate the absorbance value according to the equation

T = I/Io(1)

A = log_10_ (1/T) or A = 2 − log_10_ (%T) (2)

where T = transmittance, I = transmitted light, Io = incident light, and A = absorbance.

The absorbance value at 625 nm is used to calculate the albumin concentration according to the equation

Albumin = [(A-intercept)/slope] × Dilution factor(3)

where A is an absorbance at 625 nm and the slope and intercept are from the linear calibration.

The absorbance value at 515 nm is used to calculate the creatinine concentration according to the equation

Creatinine = [(A-intercept)/slope] × Dilution factor(4)

where A is an absorbance at 515 nm and the slope and intercept are from the linear calibration.

The albumin-to-creatinine ratio (uACR) is calculated according to the equation

uACR = (Albumin × 100)/Creatinine(5)

Results are displayed and interpreted as follows: (i) normal urinary albumin if uACR < 30 mg/g creatinine, (ii) microalbuminuria if uACR = 30–300 mg/g creatinine, and (iii) macroalbuminuria if uACR > 300 mg/g creatinine [20,21].

### 2.3. Colorimetric Detection of Albumin and Creatinine

Albumin concentrations in urine samples (artificial and clinical samples) were determined using a spectrophotometric dye-binding assay. The dye used in the reaction was tetrabromophenol blue (TBPB) [22].

Albumin + tetrabromophenol blue in acetate buffer (pH 3.2) → ALB-TBPB Complex

Concentrations of creatinine (artificial and clinical samples) were determined using the colorimetric Jaffe reaction in alkaline conditions [23,24,25].

Creatinine + Picric Acid + NaOH → CRE-Picrate Complex

The reaction mixture of creatinine was incubated at room temperature (25 °C) for 30 min, and uACR was determined using MyACR.

### 2.4. MyACR Detector Linearity

UV-visible absorbance of standard solutions of albumin (7 concentrations in the range of 0–60 mg/L) and creatinine (7 concentrations in the range of 0–2 mg/dL) following the reactions described above was recorded using MyACR at the absorbances of 625 nm and 515 nm, respectively. The measured values of both albumin and creatinine at all concentrations were within acceptable limits, i.e., ≤±10% deviation from the values measured using a laboratory spectrophotometer (PERSEE T6U UV/VIS Spectrometer, Auburn, CA, USA).

### 2.5. Measurement of Albumin and Creatinine in Urine Samples

For albumin reaction, 2 mL of the mixture of tetrabromophenol blue (TBPB, 5 × 10^−5^ M) and 1% Triton X-100 in 0.05 M acetate buffer (pH 3.2) were thoroughly mixed with a diluted urine (37.5 µL) sample in 2962.5 µL of distilled water (1:80) [22]. For the creatinine reaction, the alkaline picrate reaction mixture of picric acid (1 mL) and sodium hydroxide (1 mL) was thoroughly mixed with a diluted urine sample in distilled water (1:80) and incubated at room temperature for 30 min before analysis [23]. The optical density (OD) of the mixture of the urine sample (3 mL each) for determination of albumin and creatinine concentrations was measured against respective blank solutions in sample holders No. 1 (absorbance at 625 nm) and 2 (absorbance at 515 nm), respectively. The color intensity was corrected into Δintensity (absolute value) by subtracting the blank intensity and adjusting with the dilution factor (1:80).

### 2.6. Validation of Test Performance of MyACR

#### 2.6.1. Calibration Curves

For analysis of albumin in artificial urine samples, calibration curves were prepared through replicate analysis of seven samples (37.5 μL each) spiked with varying concentrations of albumin (0, 2, 5, 10, 20, 40, and 60 mg/L). Samples were analyzed as described above.

To analyze creatinine in artificial urine samples, calibration curves were prepared through replicate analysis of seven samples (37.5 μL each) spiked with varying concentrations of creatinine (0, 0.1, 0.25, 0.5, 1, 1.5, and 2 mg/dL). Samples were analyzed as described above.

#### 2.6.2. Accuracy

The accuracy of MyACR for determination of albumin in artificial urine samples was determined through replicate analysis of five sets of samples spiked with three different concentrations of albumin (10, 40, and 60 mg/L).

The accuracy of MyACR for the determination of creatinine in artificial urine samples was determined through replicate analysis of five sets of samples spiked with three different concentrations of creatinine (0.25, 1, and 2 mg/dL).

The accuracy of the assay method was reported as the percentage deviation of the mean value (% deviation of mean value (DMV)) from the theoretical value (laboratory spectrophotometer):

Accuracy (%) = [(mean − nominal)/nominal] × 100(6)

#### 2.6.3. Precision

The precision of MyACR for the determination of albumin in artificial urine samples based on within-day (repeatability) and day-to-day (reproducibility) variation was determined through replicate analysis of five sets of samples spiked with three different concentrations of albumin (10, 20, and 60 mg/L).

The precision of MyACR for the determination of creatinine in artificial urine samples based on within-day and day-to-day variation was determined through replicate analysis of five sets of samples spiked with three different concentrations of creatinine (0.25, 1, and 2 mg/dL).

The coefficient of variation (CV) was calculated from the ratio of standard deviation to the mean and expressed as a percentage:

% CV = standard deviation/mean × 100(7)

#### 2.6.4. Limit of Quantification

The limit of quantification (LOQ) of MyACR for the determination of albumin and creatinine in artificial urine samples was determined from the lowest concentration of albumin or creatinine (in spiked urine samples) that produced CV ≤ ±20% of spiked concentrations.

### 2.7. Specificity of Albumin Measurement in Imitating Pyuria and Hematuria Conditions

Blood samples were kindly provided by the blood bank at Thammasat Chalermprakiet Hospital. White blood cells (WBCs) and red blood cells (RBCs) were isolated using Histopaue media (Merck KGaA, Darmstadt, Germany). In the first set of experiments, WBCs were spiked into artificial urine at a concentration of 5 × 10^5^ cells/mL (the cut-off for pyuria is 10,000 cells/mL [26]). In the second set, RBCs were spiked into artificial urine at the same concentration of 5 × 10^5^ cells/mL (the cut-off for hematuria is 20,000 cells/mL [27]). Additionally, a third set was spiked with both WBCs and RBCs into artificial urine at concentrations of 5 × 10^5^ cells/mL each. The prepared samples were diluted with distilled water (DW) to a final ratio of 1:80 and then mixed with albumin reagent. All samples were prepared in triplicate, and absorbance was measured at 625 nm. Distilled water (DW) was used as the control.

### 2.8. Application of MyACR to Clinical Samples

The study was approved by the Ethics Committee, Faculty of Medicine, Thammasat University (Project number MTU-EC-OO-2-1-168/66, approval number 266/2566). The study was conducted at Thammasat Chalermprakiet Hospital and the Thammasat University Center of Excellence in Pharmacology and Molecular Biology of Malaria and Cholangiocarcinoma, following the guidelines outlined in the Declaration of Helsinki. Written informed consent was obtained from all subjects before study participation.

Random urine samples (2–3 mL) were collected from 20 CKD patients. All samples were stored at −20 °C until analysis. Concentrations of albumin and creatinine in urine samples were measured as described above (single measurement for each patient), and the uACR values of each sample were determined using the MyACR device and compared with a laboratory spectrophotometer and a central (hospital) laboratory method (immune-based assay). The microalbumin method is based on particle-enhanced turbidimetric inhibition immunoassay. The creatinine method measures creatinine enzymatically by involving the addition of creatininase, creatinase, and sarcosine oxidase [28].

### 2.9. Statistical Analysis

Statistical analysis was performed using SPSS 17.0 software. Quantitative variables are summarized as median (range) values. Deviation from the mean of the uACR values measured using MyACR and those measured using the standard spectrophotometer were calculated and are expressed as %DMV (% deviation from mean value). The correlation between the uACR values measured using both equipment was determined using Spearman’s correlation test at a statistical significance level of α = 0.05.

## 3. Results

### 3.1. Validation of Test Performance of MyACR Device

#### 3.1.1. Calibration Curves

**Figure 3:**
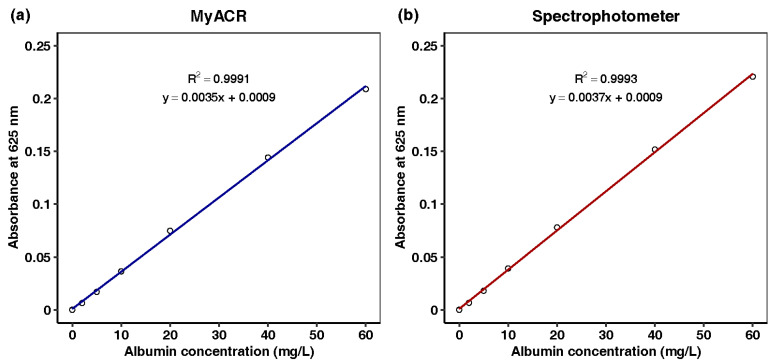
Calibration curves of albumin at the concentration range of 0–60 mg/dL determined using (**a**) MyACR and (**b**) a laboratory spectrophotometer.

**Figure 4:**
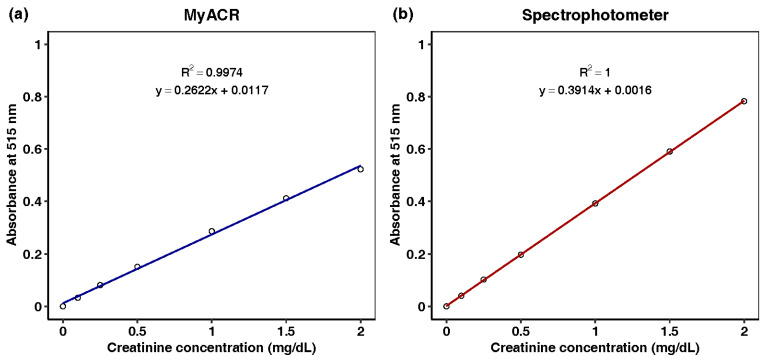
Calibration curves of creatinine at the concentration range of 0–2 mg/dL determined using (**a**) MyACR and (**b**) a laboratory spectrophotometer.

Urine analyses of albumin and creatinine using MyACR were calibrated using the concentration ranges of 0–60 mg/L and 0–2 mg/dL, respectively. All calibration ranges yielded linear relationships with correlation coefficients (R^2^) of 0.997 or better (Figure 3a,b and Figure 4a,b).

#### 3.1.2. Accuracy

Good accuracy was observed from both the intra- and inter-day assays for determination of albumin and creatinine using MyACR, as indicated by the minimal deviation of mean values found with measured samples from those of the theoretical values (actual amount added), with a %MDV of lower than ≤5.42% at all investigated concentrations (Table 2 and Table 3).

#### 3.1.3. Precision

Acceptable variation (intra- and inter-assay variation) of albumin and creatinine assays in artificial urine samples was observed with CV ≤ 12.69% at all investigated concentrations (Table 2 and Table 3).

#### 3.1.4. Limit of Quantification

The LOQ of albumin and creatinine in urine samples determined using MyACR and a laboratory spectroscopy with %CV ≤ 20% were 5 mg/L and 0.25 mg/dL, respectively, using 37.5 µL urine samples.

### 3.2. Specificity of Albumin Measurement in Imitating Pyuria and Hematuria Conditions

The absorbances of samples, including the artificial urine spiked with 5 × 10^5^ WBCs/mL, 5 × 10^5^ RBCs/mL, and a combination of WBCs and RBCs at a concentration of 5 × 10^5^ cells/mL each, are presented in Table 4. All conditions showed very slight differences from the distilled water control.

### 3.3. Application to Clinical Samples

**Figure 5:**
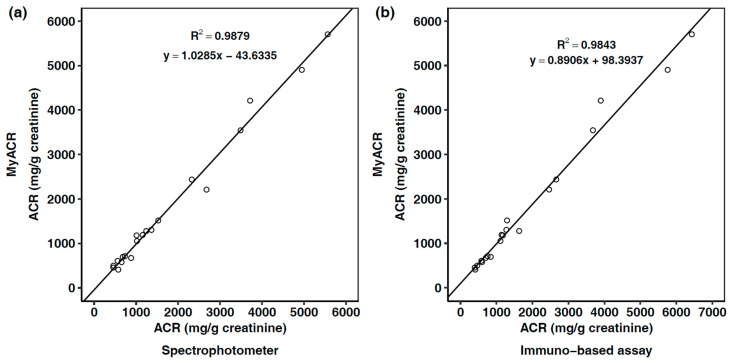
Correlation between uACR measured using (**a**) MyACR vs. laboratory spectrophotometer and (**b**) MyACR vs. immune-based assay (gold standard) from 20 subjects. The reactions used for albumin and creatinine measurement using MyACR and a laboratory spectrophotometer are the dye (tetrabromophenol blue)-binding assay and the colorimetric Jaffe assay, respectively. The reactions used for albumin and creatinine measurement in the central (hospital) laboratory are an immune-based assay and an enzymatic method, respectively.

To evaluate the clinical applicability of MyACR, the uACR values in urine samples of 20 CKD patients measured using MyACR were compared with those measured using the laboratory spectrophotometer and central (hospital) laboratory (immune-based assay) methods. The median (range) of %DMV of the spectrophotometer and immune-based method were −1.88 (−16.88 to 28.98)% and 3.48 (−17.05 to 21.64)%, respectively. The correlation of the values measured between the two methods was high, with an R^2^ of ≥0.98 (Figure 5a,b).

## 4. Discussion

Of all proteins, albumin, synthesized in the liver, is very important as an active substance in the human body. In healthy individuals, the albumin content in urine is typically less than 30 mg/g creatinine, and any value higher than this may indicate potential kidney disease [29]. Creatinine is the ultimate metabolite of nitrogen elements in the human body, primarily excreted from the blood by the kidneys, specifically through glomerular filtration. Low urinary excretion of creatinine reflects impairment of kidney function and corresponding renal diseases [30].

The use of the urine (random) albumin-to-creatinine ratio (uACR) is currently recommended as the preferred screening strategy for determining the risk and monitoring the progress of CKD [31]. The standard method for uACR determination involves measuring albumin and creatinine separately using automated machines. The available analytical techniques used for detecting each substance are complex, time-consuming, and expensive, and they require modern and less portable instruments, high-cost analyses, large samples and reagent volumes, and the services of skilled medical technicians. Due to the low concentration of albumin in urine, it is difficult to measure the concentration level using a spectrophotometer and colorimetric methods [32], and more specific methods, such as immunoturbidimetry [33,34], immunoassays [35,36], radioimmunoassay [37], and liquid chromatography, have been applied [38]. In addition, several sensitive methods have been developed to determine microalbuminuria and/or uACR. These include paper-based analytical devices (PADs) [10,39,40,41,42,43], microfluidic sliding PAD [44], sequential injection analysis (SIA) [45,46], and the mono-segmented sequential injection lab-at-valve (SI-LAV) system [47]. Although the performance of some tests is considered good, they are time-consuming and require complicated devices or external tools, such as pumps and high-voltage machines, which are impractical for PoCT. Therefore, developing a simple device with a short analysis time is needed to be portable and affordable for the PoCT of CKD.

Research articles on developing PoCT devices for uACR measurement have highlighted technological advances, biosensor-based approaches, microfluidic systems, smartphone-based technologies, clinical validation studies, and challenges in translation and implementation. Biosensors typically involve immobilizing specific antibodies or aptamers on a sensor surface to capture albumin, followed by detecting creatinine or albumin–creatinine complexes. Such biosensors offer the advantages of rapid analysis, minimal sample volume requirements, and potential for PoCT. However, they may be susceptible to interference from matrix components or substances in biological samples, leading to false-positive or false-negative results. Some biosensors may exhibit limited sensitivity, particularly when detecting low concentrations of albumin or creatinine in urine samples [48]. Microfluidic-based PoCT devices have gained attention for their ability to perform complex sample handling and analysis tasks on a miniaturized platform [10,11,39,42]. Microfluidics have been integrated with sensing elements for uACR measurement, enabling precise control of sample flow and reaction kinetics. These systems may, however, encounter challenges related to sample handling, such as sample evaporation, air bubble formation, and clogging of microchannels. Smartphone-based PoCT devices have emerged as promising platforms for decentralized testing [49,50,51]. These devices leverage smartphones’ computational power and connectivity to perform uACR analysis using built-in cameras, sensors, and dedicated mobile applications. The measurement modes include colorimetric assay, fluorescence detection, and paper-based microfluidics. Smartphone-based assays may face compatibility issues with different smartphone models or operating systems, limiting their universal applicability.

The PoCT devices commercially available for determining uACR are immune-based assays. These include the DCA Vantage Analyzer (Siemens, Munich, Germany), Afinion^TM^ AS100 Analyzer (Abbotts, Green Oaks, IL, USA), NycoCard^TM^ READER II (Abbotts Rapid Diagnostics, Orlando, FL, USA), and QuikRead go^TM^ Instrument (Orion Diagnostica, Espoo, Finland) [52]. While PoCT devices offer numerous advantages for uACR measurement, including rapid results, point-of-care testing, minimal sample volume, ease of use, and portability, they also present limitations related to cost, quality control, interference, regulatory compliance, and data management.

In this study, we developed MyACR as a PoCT for screening the risk and monitoring the progress of CKD using uACR as a biomarker. The device is portable, user-friendly, accurate, reproducible, and cost-effective, with comparable performance to a laboratory spectrophotometer. The analysis runtime is relatively short, with a reaction time of 30 min. Our device is capable of simultaneously measuring creatinine and albumin, resulting in an analysis time of less than 1 min. The test procedure is also simple. Its potential benefit for community healthcare facilities with limited laboratory resources needs further evaluation. Unskilled users can operate MyACR, and minimal to no auxiliary equipment is needed. The methods are based on colorimetric dye-forming reactions, in which the optical density of the color complex formed from each reaction is measured using visible UV at wavelengths of 515 nm (for creatinine) and 625 nm (for albumin). Moreover, the proposed system utilizes the slimmer cuvettes (12.5 × 7.5 × 45 mm) rather than the conventional cuvettes (12.5 × 12.5 × 45 mm) in a spectrophotometer with the same pathlength (10 mm) to reduce the measuring solution volume. The device can simultaneously measure both compounds and provide the uACR for each sample. The Jaffe reaction is the most commonly used method for creatinine determination, relying on the formation of an absorbing complex during the reaction of creatinine with picric acid. Several dyes have been used in the albumin reaction, including bromocresol green and bromocresol purple [53], bromophenol blue [54], tetrabromophenol blue [55], bromochlorophenol blue [56], Commassie Brillant Blue G [57], and eosin [58] dyes. Calibration curves for both compounds can be prepared only once when the device is first used, and the information is recorded in the device database. uACR can be measured in a single random urine sample without the requirement of batch analysis. The accuracy and reproducibility of both assay methods are within acceptable ranges (%DVM ≤ 5.42%, %CV ≤ 12.69%). Although the methods are based on spectroscopic assays, the sensitivity of the albumin and creatinine analyses is within an acceptable range. It is adequate to detect microalbuminuria (LOQ) of 5 mg/L and 0.25 mg/dL for albumin and creatinine, respectively. Due to the low sensitivity of albumin, we consider the lowest concentration that provided accuracy and precision within ±20% to be the LOQ. This criterion is based on the ICH guidelines, and the acceptance criteria for accuracy and precision at low concentrations should be within ±20% [59]. Quality control (QC) samples (with three concentrations, low, medium, and high) should be integrated into the analysis at least once every two weeks. An acceptable deviation of uACR values from each QC sample is ±5%. New calibration curves are required to accurately analyze uACR in the case of deviation outside of this acceptable limit. In addition, glomerulonephritis as well as pyelonephritis, which are abnormal kidney conditions, can present with pyuria or hematuria. To confirm the specificity of the reaction method to only albumin, artificial urine spiked with WBCs, RBCs, and a combination of WBCs and RBCs was analyzed. The absorbance results showed very slight differences in the absorbance of the spiked samples compared to the distilled water control. The concentration of WBCs and RBCs used was 5 × 10^5^ cells/mL, which is higher than the cut-off for pyuria (10,000 cells/mL [26]) and hematuria (20,000 cells/mL [27]) conditions. The clinical applicability of MyACR to determine uACR was well-demonstrated in urine samples collected from 20 CKD patients. An excellent correlation (r^2^ > 0.98) was found between the values measured by the device compared with the laboratory spectrophotometer and the gold standard method used in the central (hospital) laboratory (immune-based assay).

While PoCT devices offer numerous advantages for uACR measurement, including rapid results, point-of-care testing, minimal sample volume, ease of use, and portability, they also present limitations related to cost, quality control, interference, regulatory compliance, and data management.

## 5. Conclusions

MyACR, a simple platform based on colorimetric detection for the determination of uACR, was developed for use as a PoCT for determining the risk and monitoring the progress of CKD. The device showed satisfactory accuracy, reproducibility, sensitivity, and specificity test performance. Cost-effectiveness and improvement in clinical decision making need to be proven in future multisite community and home studies. Validation of the clinical application of the device in a large sample size, including healthy subjects, CDK patients, and different groups of patients at risk of CKD (particularly those with diabetes and hypertension), is underway.

## 6. Patents

The innovation patent ‘MyACR’ is approved by the Department of Intellectual Property of Thailand (No. 2302005386).

## Figures and Tables

**Table 1:** Composition of artificial urine used to evaluate the test performance of MyACR [19].

Composition	Molecular Formula	Molecular Weight (g/mol)	Final Concentration (mM)
Sodium sulfate anhydrous	Na_2_SO_4_	142.04	9
Trisodium citrate dihydrate	Na_3_C_6_H_5_O_7_·2H_2_O	294.1	5
Urea	CH_4_N_2_O	60.06	200
Potassium chloride	KCl	74.55	30
Sodium chloride	NaCl	58.44	54
Calcium chloride	CaCl_2_	110.99	3
Ammonium chloride	NH_4_Cl	53.49	15
Magnesium sulfate heptahydrate	MgSO_4_·7H_2_O	246.47	2
Sodium phosphate monobasic monohydrate	NaH_2_PO_4_·H_2_O	137.99	3.6
Sodium phosphate dibasic heptahydrate	Na_2_HPO_4_·7H_2_O	268.03	0.4
Sodium bicarbonate	NaHCO_3_	84.01	2
Disodium oxalate	Na_2_C_2_O_4_	134	0.1

**Table 2:** Intra- and inter-assay precision and accuracy of albumin assay determined using MyACR compared with a laboratory spectrophotometer.

Albumin Concentration Added (mg/L)	Precision (%CV)		Accuracy (%DVM)	
	Intra-Day	Inter-Day	Intra-Day	Inter-Day
MyACR				
10	2.52	4.58	1.43	4.29
40	1.34	3.02	2.36	1.07
60	1.44	2.75	−0.90	−1.57
Spectrophotometer				
10	2.86	3.77	3.51	6.22
40	0.30	1.00	1.96	2.50
60	1.42	0.87	−0.95	−0.95

**Table 3:** Intra- and inter-assay precision and accuracy of creatinine assay using MyACR compared with a laboratory spectrophotometer.

Creatinine Concentration Added (mg/L)	Precision (%CV)		Accuracy (%DVM)	
	Intra-Day	INTER-DAY	Intra-Day	Inter-Day
MyACR				
0.25	1.21	12.69	5.42	−2.82
1	1.08	4.54	4.77	1.03
2	1.35	3.53	−2.57	−4.14
Spectrophotometer				
0.25	1.99	2.35	2.61	3.83
1	1.25	2.32	−0.15	1.43
2	1.42	2.40	−0.20	1.23

**Table 4:** Specificity of albumin measurement in imitating pyuria and hematuria conditions.

Conditions	Absorbance at 625 nm [Median (Range)]	
	Spectrophotometer	MyACR
Artificial urine spiked with distilled water (control)	0 (0 to 0)	0 (0 to 0)
Artificial urine spiked with WBCs	0 (0 to 0.001)	−0.003 (−0.003 to 0)
Artificial urine spiked with RBCs	0.001 (0.001 to 0.002)	−0.003 (−0.005 to −0.002)
Artificial urine spiked with WBCs and RBCs	0.002 (0.002 to 0.002)	0.004 (−0.005 to −0.003)

## Data Availability

Data are contained within the article.
